# Psychosocial issues and concerns of cancer patients due to COVID-19 pandemic lockdown

**DOI:** 10.1371/journal.pgph.0000996

**Published:** 2022-09-09

**Authors:** Surendran Veeraiah, Sundaramoorthy Chidambaram, Revathy Sudhakar, Selvaluxmi Ganesharaja, Arvind Krishnamurthy, Manikandan Dhanushkodi, Rajaraman Swaminathan

**Affiliations:** 1 Department of Psycho-Oncology & RCTC, Cancer Institute (WIA), Chennai, India; 2 Department of Radiation Oncology, Cancer Institute (WIA), Chennai, India; 3 Department of Surgical Oncology, Cancer Institute (WIA), Chennai, India; 4 Department of Medical Oncology, Cancer Institute (WIA), Chennai, India; 5 Department of Epidemiology, Bio-Statistics and Tumor Registry, Cancer Institute (WIA), Chennai, India; PLOS: Public Library of Science, UNITED STATES

## Abstract

**Background:**

The COVID-19 pandemic lockdown has posed numerous unique challenges for cancer patients, families and healthcare workers. However, the reports on psychosocial issues associated with such situations are scarce. This study aims to determine the psychosocial issues faced by cancer patients during COVID-19 pandemic lockdown.

**Methods:**

Cancer patients irrespective of diagnosis and treatment status were assessed for fear of progression (FOP), distress and quality of life (QOL) using Fear of Progression- Short Form, Distress Thermometer and European Organization for Research and Treatment of Cancer Quality of Life Questionnaire (EORTC QLQ C30) 30, respectively. The demographics, disease and treatment related details were obtained from case record form. Psychological issues and concerns were collected using a structured interview. Descriptive statistics, Mann Whitney U-test and Linear Regression were performed using SPSS ver 20.0.

**Results:**

Among the 219 patients, 118 (52.5%) had either interruption in their on-going cancer treatment or the initiation of cancer treatment was delayed as a result of COVID-19 lockdown. Overall, 74% of the patients experienced distress, 55.3% experienced FOP and 58% had low global health status. Pain followed by fatigue remained as major issues among patients during lockdown. Interruption in treatment and logistical issues were strongly associated with increased distress (p = 0.026) and FOP (p = 0.004). Global health status (p = 0.037), emotional functioning (p = 0.000), social functioning (p = 0.000) and financial concerns (p = 0.046) differed significantly between patients with and without treatment interruption. Age (β = -0.159), mode of transport (β = -0.135), challenges in meeting daily needs (β = -0.245) and being out-casted by the society (β = -0.227) predicted distress.

**Conclusion:**

More than half of the patients had interruptions in their treatment as a result of COVID-19 lockdown. Cancer patients have had increased physical and psychological concerns as a result of the pandemic situation and its associated changes. Specific guidelines ought to be framed for providing continued and holistic cancer care for patients during such lockdown.

## Introduction

The sudden onset of COVID-19 pandemic posed serious physical and psychological impact on cancer patients and their families. Cancer patients are at high risk of serious illness from an infection because their immune system are often weakened by cancer and its treatment [1]. Cancer is among one of the more serious comorbidities that increase risks of Corona virus diseases (COVID-19). Patients with lymphohematologic malignancies, receiving chemotherapy or intensive radiotherapy, monoclonal antibody treatments or other targeted treatments such as protein kinase inhibitors, or who have undergone bone marrow or stem cell transplants in the last six months, are particularly vulnerable as these treatments significantly weaken their immune system [1]. To protect the immune-compromised individuals, oncologists, and health-care workers, considering the individual patient history, stage of cancer, specific treatment plan and then weigh the risks of delaying versus providing treatment is mandated [2]. The rapid and global spread of the virus has forced governments to take unprecedented measures to restrict travel and contact between individuals, as well as restrict access to hospitals for medically unavoidable patients only to reduce straining health systems [1]. Since March 2020, guidelines on cancer treatment and risk-stratified care had started emerging in India, suggesting modification or deferment of treatment, if considered safe.

In addition to the nature of the disease and treatment, the constant news about COVID-19 can be extremely worrying for cancer patients and their families. Travel restrictions, patient concerns, regulatory guidance, and re-allocating of oncology staff have resulted in many cancer outpatient visits being replaced by telephone consultation, and deferral of some routine therapy, tests, and procedures [3]. Although many centres continued to offer cancer treatment, nearly 70% of patients could not access life-saving surgeries and treatment, chemotherapy treatment and follow-ups were postponed. Overall cancer services declined by 50% in April and May 2020 and at least 51,100 life-saving cancer surgeries were cancelled in India from the end of March to the end of May 2020 [4]. All these scenario have caused increased psychological distress among cancer patients and their families. The uncertainty about treatment continuation due to the virus spread was found to be more stressful for patients even for the newly diagnosed [2].

Although many studies are being conducted around pandemic situation in oncology across many countries, only few focus upon the psychosocial impact of COVID-19 pandemic and concerns of patients themselves during this circumstance, particularly in India. Hence, the psychosocial concerns and well-being of patients and their families are unknown at present. As the lifestyle modifications taking place in line with the pandemic situation is the proposed new normal, the results of this study could help in tailoring appropriate psychosocial intervention to cater to the needs of cancer patients and their families during such scenario at present and in future. Among adult cancer patients visiting Cancer Institute (WIA), a regional comprehensive cancer centre, to address the gaps discussed above, this study aims to assess the psychosocial issues and concerns during COVID-19 pandemic.

The specific objectives of the study were to study the level of distress, FOP, coping and its association with QOL among cancer patients during COVID-19 pandemic, to compare the levels of distress, FOP, coping, QOL of cancer patients treated before and after COVID-19 pandemic onset and to study physical, psychological, logistical, social and informational concerns among cancer patients treated before and after COVID-19 pandemic onset.

## Methods

### Study design

This study employed a cross-sectional study design.

### Setting

Tamil Nadu (TN) is the sixth largest state by population in India inhabited by nearly 72 million people and experienced a huge impact of COVID-19 with strict restrictions. Around 8.5 lakh COVID cases had been registered in Tamil Nadu since March 2020 and lockdown was enforced on 24^th^ March 2020. The Cancer Institute (WIA) is a comprehensive, public cancer centre comprising of 535 beds, a Research Division, a College of Oncological Sciences, and a division of Preventive Oncology. It also houses a separate pain and palliative care unit and a hospice catering to the needs of advanced cancer patients as well. All the patients diagnosed with cancer before/during COVID-19 pandemic and with/without treatment interruption during the COVID-19 pandemic lockdown and visiting the OPDs on/after August 2020 were included for the study.

### Study population

All adult cancer patients above the age of 18 years, irrespective of the diagnosis and stage of the disease who visited Cancer Institute (WIA) from August to October 2020 were considered eligible for the study. The demographic (age, gender, socio-economic status, education, occupation, family details) and other details regarding patients’ diagnosis, treatment status was obtained from the hospital-based cancer registry.

### Data variables, sources of data and data collection

The data was collected from the adult cancer patients, above 18 years of age reporting to the out-patient departments (Medical/Radiation/Surgical Oncology) during their visit to the institute and from eligible patients through telephone, due to travel restrictions. The participants were initially briefed by the Psycho-oncologist about the purpose of the survey along with an information sheet and written consent was obtained for data collection. National Comprehensive Cancer Network distress thermometer [5] was used for assessing distress which was categorized as mild, moderate and severe distress, Cancer Coping Questionnaire [6] consisting of individual scale and interpersonal scale for assessing levels of coping, Fear of Progression Questionnaire- Short form [7] for assessing higher or lower levels of FOP, European Organization for Research and Treatment for Cancer C30 [8] for assessing the QOL and structured interview schedule for assessing the psychosocial issues and concerns namely physical, psychological, social, informational and financial. All the assessments were administered by the primary researcher who was a qualified Psycho-oncologist trained in conducting researches. The assessments carried out only one time for each patient and the average duration of each interview was around 30 minutes. The demographics, disease and treatment related details were obtained from the hospital case record form. The responses were recorded in a separate response sheet.

### Operational definition

#### Psychosocial issues

Issues caused by environmental and/or biological factors on individual’s social and/or psychological aspects [9].

#### Distress

A multifactorial unpleasant emotional experience of a psychological (cognitive, emotional, behavioural) social, and/or spiritual nature that may interfere with the ability to cope effectively with cancer at the time of COVID-19 pandemic [10].

#### Coping

An individual’s cognitive and behavioural efforts to manage stress associated with cancer and treatment during COVID-19 pandemic [11].

#### Fear of progression

Patient’s fear that the illness will progress with all its biopsychosocial consequences or that it will occur, during pandemic [12].

### Sampling

All cancer patients irrespective of the diagnosis and stage of the disease, visiting the out-patient department at Cancer Institute (WIA), Chennai from August to October 2020 were recruited.

### Statistical analysis

The data collected was double entered and validated using Statistical Services for the Social Sciences version 20. Participant characteristics and key outcome indicators such as Distress, FOP and QOL domains were summarized using frequency analysis. The physical, psychological, logistical and social issues were represented using numbers and proportions. A distress score of >4 was considered as significant distress and <4 as no significant distress. The scores of FOP and QOL domains were categorized using mean score into below and above mean for statistical purpose. Chi-square test was used to examine the association between the socio-demographic variables and distress, FOP and global health status as well as to examine the association between the psychosocial issues with distress, FOP and treatment status. Mann-Whitney U test was used to find difference between different treatment status (Interrupted /Delayed vs. Uninterrupted) and distress, FoP, QOL domains. The effect size estimate of between-group was calculated using the formula, r = Z/√ N and classified as follows: 0.1–0.3 small effect; 0.3–0.5 inter mediate effect; and ≥0.5, strong effect [13]. Stepwise multiple linear regressions were performed separately for distress, FOP and QOL domains to find the sociodemographic predictors and psychosocial issues that act as predictors. The variables were first subjected to univariate analysis. All significant variables were then subjected to multivariate analysis. Each variable was adjusted for the rest in the final model. β coefficient was used to express the relationship of variables in the study. The F value at *p* = 0.05 and p = 0.10 were considered as stepping method criteria for entry and for removal, respectively. The significance level for all analyses was considered at p < 0.05 and two-tailed.

#### Ethics approval

Ethics approval was obtained from the Institutional Ethics Committee, Cancer Institute (WIA), Chennai, Tamil Nadu. Written consent from the participants was obtained prior to the interview.

#### Data confidentiality

Hardcopies of data sheets were kept confidentially in a locked cabinet accessible only to the study investigators at the Department of Psycho-oncology, Cancer Institute (WIA). Electronic data was stored in password protected computers.

## Results

Of the 279 patients who reported to the out-patient department at the institute during the study period and contacted over telephone, 219 patients completed the survey. Among the 219 valid respondents, 118 (52.5%) patients had either interruption in their on-going cancer treatment or the initiation of cancer treatment was delayed as a result of COVID-19 lockdown, whereas 101 (47.5%) patients had no disruptions in their treatment. More than half of the patients were between the age group 46–65 years. Most of the respondents (136; 62.1%) were female and majority (185; 84.5%) were married. While 40.2% had completed secondary/ higher secondary level of education, majority (96; 43.8%) were from low socio-economic status. Around 60.7% of the patients were with a stage III/IV disease. More than three fourth of the patients were aware of their diagnosis (178; 81.3%) and more than half, of their prognosis (140; 63.9%). Very few patients (20; 9.1%) had been diagnosed with and treated for COVID-19 (Table 1).

**Table 1:** Personal and clinical characteristics of study participants (n = 219).

Characteristics	Category	Frequency	Percentage
Age	Mean age in Years (SD)	50.12 (11.24)	
	Range	20–81	
	18–45	76	34.7
	46–65	124	56.6
	Above 65	19	8.7
Gender	Male	83	37.9
	Female	136	62.1
Marital status	Married	185	84.5
	Single/ Widowed/separated	34	15.5
Education	Illiterate	40	18.3
	Primary	61	27.9
	Secondary/Higher Secondary	88	40.2
	Graduate	30	13.7
Occupation	Working	82	37.4
	Not working	137	62.6
Income (INR)	< 5000	96	43.8
	5001–10000	76	34.7
	>10000	47	21.5
Residence	Rural	137	62.6
	Urban	82	37.4
Mode of Transport used	Own Vehicle	88	40.2
	Private (Hired)	126	57.5
	Govt.	5	2.3
Diagnosis	Breast	73	33.3
	Head and Neck	55	25.1
	Gynecology	37	16.9
	Other Can	34	15.5
	Digestive/Gastrointestinal	20	9.1
Stage	I/II	86	39.3
	III/IV	133	60.7
Treatment status	Interruption/delayed	118	52.5
	No Interruption/Completed	101	47.5
Awareness of Diagnosis	Completely Aware	178	81.3
	Partially Aware/Unaware	41	18.7
Awareness of Prognosis	Completely Aware	140	63.9
	Partially Aware/Unaware	79	36.1
Patients affected by COVID-19	Yes	20	9.1
	No	199	90.9

Pain (96; 43.8%), followed by fatigue (82; 37.4%) remained as major issues among patients during the pandemic lockdown. Majority of the patients reported distress (161; 74%), while fear, anxiety, worry and sleep difficulty were also reported to a great extent. Nearly half reported difficulty in commuting to the hospital during the lockdown and more than one-third reported difficulty in getting medical access. Nearly 20% reported difficulty in obtaining e-pass. Majority had support from their family (177; 80.8%), friends (162; 74%) and government (125; 57.1%). Less than half reported to have received informational support during the lockdown.

Overall, 74% of the patients experienced distress, 55.3% experienced FOP and 58% had low global health status. Participants reported pain (43.8%), fatigue (37.4%), feeling anxious (58.4%), worry (52.5%), sleep problems (36.5%), travelling issues (46.6%), difficulty in getting medical care (40.2), E-pass issues (19.2), accommodation problems (22.8%), financial issues (71.7%), difficult in managing daily needs (38.8%), poor informational support (44.3%) during COVID-19 lockdown.

Treatment status (p = 0.022), awareness of diagnosis (p = 0.014), mode of transport used to commute to the hospital (p = 0.000), logistical issues, social issues and the level of FOP (p = 0.004) were significantly associated with distress. Education (p = 0.000), awareness of diagnosis (p = 0.004) and prognosis (p = 0.018), been diagnosed with COVID-19 (p = 0.020), social functioning (p = 0.016) difficulty in transportation (p = 0.019) and accommodation (0.039), meeting daily needs (p = 0.000) and familial support (p = 0.007) were significantly associated with FOP (Tables 2 and 3). Similarly, dimensions of functional scale and symptom scale in addition to the accessibility to medical services (p = 0.000) were found to be significantly associated with global health status (Table 3).

**Table 2:** Association of personal and clinical characteristics with distress, FOP and global health status during COVID-19 lockdown.

Variables	Category	Total Score	Distress			Fear of Progression (Fop)			Global Health Status		
			<4 DT Score	>4 DT score	*P-Value*	Below average	Above average	*P-Value*	Below average	Above average	*P-Value*
			n (%)	n (%)		n (%)	n (%)		n (%)	n (%)	
Age (Years)	18–45	76	46 (60.5)	30 (39.5)	.058	38 (50.0)	38 (50.0)	.465	43 (56.6)	33 (43.4)	.873
	46–65	124	80 (64.5)	44 (35.5)		53 (42.7)	71 (57.3)		72 (58.1)	52 (41.9)	
	Above 65	19	17 (89.5)	2 (10.5)		7 (36.8)	12 (63.2)		12 (63.2)	7 (36.8)	
Gender	Male	83	59 (71.1)	24 (28.9)	.160	39 (47.0)	44 (53.0)	.603	45 (54.2)	38 (45.8)	.377
	Female	136	84 (61.8)	52 (38.2)		59 (43.4)	77 (56.6)		82 (60.3)	54 (39.7)	
Marital status	Married	185	121 (65.4)	64 (34.6)	.937	81 (43.8)	104(56.2)	.503	109 (58.9)	76 (41.1)	.516
	Single/ Widowed/ separated	34	22 (64.7)	12 (35.3)		17 (50.0)	17 (50.0)		18 (52.9)	16 (47.1)	
Education	Illiterate	40	24 (60.0)	16 (40.0)	.887	7 (17.5)	33 (82.5)	.000***	24 (60.0)	16 (40.0)	.088
	Primary	61	40 (65.6)	21 (34.4)		25 (41.0)	36 (59.0)		43 (70.5)	18 (29.5)	
	Secondary/Higher Secondary	88	59 (67.0)	29 (33.0)		46 (52.3)	42 (47.7)		44 (50.0)	44 (50.0)	
	Graduate	30	20 (66.7)	10 (33.3)		20 (66.7)	10 (33.3)		16 (53.3)	14 (46.7)	
Occupation	Working	82	54 (65.9)	28 (34.1)	.893	36 (43.9)	46 (56.1)	.845	47 (57.3)	35 (42.7)	.876
	Not working	137	89 (65.0)	48 (35.0)		62 (45.3)	75 (54.7)		80 (58.4)	57 (41.6)	
Income (INR)	< 5000	96	60 (62.5)	36 (37.5)	.501	41 (42.7)	55 (57.3)	.863	58 (60.4)	38 (39.6)	.674
	5001–10000	76	49 (64.5)	27 (35.5)		35 (46.1)	41 (53.9)		41 (53.9)	35 (46.1)	
	<10000	47	34 (72.3)	13 (27.7)		22 (46.8)	25 (53.2)		28 (59.6)	19 (40.0)	
Type of Family	Joint	81	55 (67.9)	26 (32.1)	.535	31 (38.3)	50 (61.7)	.140	41 (50.6)	40 (49.4)	.090
	Nuclear	138	88 (63.8)	50 (36.2)		67 (48.6)	71 (51.4)		86 (62.3)	52 (37.7)	
Residence	Rural	137	86 (62.8)	51 (37.2)	.311	62 (45.3)	75 (54.7)	.845	84 (61.3)	53 (38.7)	.198
	Urban	82	57 (69.5)	25 (30.5)		36 (43.9)	46 (56.1)		43 (52.4)	39 (47.6)	
Diagnosis	Head and Neck	55	40 (72.7)	15 (27.3)	.743	21 (38.2)	34 (61.8)	.497	37 (67.3)	18 (32.7)	.315
	Digestive/ Gastrointestinal	20	12 (60.0)	8 (40.0)		10 (50.0)	10 (50.0)		11 (55.0)	9 (45.0)	
	Gynecology	37	23 (62.2)	14 (37.8)		16 (43.2)	21 (56.8)		22 (59.5)	15 (40.5)	
	Breast	73	47 (64.4)	26 (35.6)		38(52.1)	35 (47.9)		42 (57.5)	31 (42.5)	
	Other Cancers	34	21 (61.8)	13 (38.2)		13 (38.2)	21(61.8)		15 (44.1)	19 (55.9)	
Stage	I/II	86	62 (72.1)	24 (27.9)	.089	43 (50.0)	43 (50.0)	.209	48 (55.8)	38 (44.2)	.600
	III/IV	133	81 (60.9)	52 (39.1)		55 (41.4)	78 (58.6)		79 (59.4)	54 (40.6)	
Treatment status	Interrupted/ delayed	118	69 (58.5)	49 (41.5)	.022*	46 (39.0)	72 (61.0)	.064	75 (63.6)	43 (36.4)	.071
	No interruption/ completed	101	74 (73.3)	27 (26.7)		52 (51.5)	49 (48.5)		52 (51.5)	49 (48.5)	
Awareness of Diagnosis	Completely Aware	178	123 (69.1)	55 (30.1)	.014*	88 (49.4)	90 (50.6)	.004**	105 (59.0)	73 (41.0)	.533
	Partially Aware/ Unaware	41	20 (48.8)	21 (51.2)		10 (24.4)	31 (75.6)		22 (53.7)	19 (46.3)	
Awareness of Prognosis	Completely Aware	140	94 (67.1)	46 (32.9)	.445	71 (50.7)	69 (49.3)	.018*	82 (58.6)	58 (41.4)	.817
	Partially Aware/ Unaware	79	49 (62.0)	30 (38.0)		27 (34.2)	52 (65.8)		45 (57.0)	34 (43.0)	
Affected with COVID-19	Yes	20	10 (50.0)	10 (50.0)	.132	4 (20.0)	16 (80.0)	.020*	14 (70.0)	16 (30.0)	.254
	No	199	133 (66.8)	66 (33.2)		94 (47.2)	105 (52.8)		113 (56.8)	86 (43.2)	
Mode of Transport used	Own vehicle	88	71 (80.7)	17 (19.3)	.000***	42 (47.7)	46 (52.3)	.762	46 (52.3)	42 (47.7)	.262
	Private (Hired)	126	68 (54.0)	58 (46.0)		54 (42.9)	72 (57.1)		77 (61.1)	49 (38.2)	
	Govt.	5	4 (80.0)	1 (20.0)		2 (40.0)	3 (60.0)		4 (80.0)	1 (20.0)	

*p<0.05,

**p<0.01,

***p<0.001.

DT = Distress thermometer score.

**Table 3:** Association of psychosocial concerns and issues of study participants (n = 219) with distress, FOP and global health status during COVID-19 lockdown.

Variables	Category	Total	Distress			Fear of progression (FOP)			Global Health Status		
			<4 DT Score	>4 DT score	P value	Below average	Above average	P value	Below average	Above average	P value
			n (%)	n (%)		n (%)	n (%)		n (%)	n (%)	
Functional Scale											
Physical functioning	Below average	101	65 (64.4%)	36 (35.6%)	0.787	41 (40.6%)	60 (59.4%)	0.253	70 (69.3%)	31 (30.7%)	0.002**
	Above average	118	78 (66.1%)	40 (33.9%)		57 (48.3%)	61 (51.7%)		57 (48.3%)	61 (51.7%)	
Role functioning	Below average	135	82 (60.7%)	53 (39.2%)	0.073	63 (46.7%)	72 (53.3%)	0.469	77 (57.0%)	58 (43.0%)	0.717
	Above average	84	61 (72.6%)	23 (27.2%)		35 (41.7%)	49 (58.3%)		50 (59.5%)	34 (40.5%)	
Emotional functioning	Below average	87	51 (58.6%)	36 (41.4%)	0.092	36 (41.4%)	51 (58.6%)	0.416	60 (69.0%)	27 (31.0%)	0.008**
	Above average	132	92 (69.7%)	40 (30.3%)		62 (47.0%)	70 (53.0%)		67 (50.8%)	65 (49.2%)	
Cognitive functioning	Below average	63	45 (71.4%)	18 (28.6%)	0.226	26 (41.3%)	37 (58.7%)	0.511	47 (74.6%)	16 (25.4%)	0.002**
	Above average	156	98 (62.8%)	58 (37.2%)		72 (46.2%)	84 (53.8%)		80 (51.3%)	76 (48.7%)	
Social functioning	Below average	129	81 (62.8%)	48 (37.2%)	0.351	49 (38.0%)	80 (62.0%)	0.016	94 (72.9%)	35 (27.1%)	0.000***
	Above average	90	62 (68.9%)	28 (31.1%)		49 (54.4%)	41 (45.6%)		33 (36.7%)	57 (63.3%)	
Symptom scale											
Fatigue	Below average	118	74 (62.7%)	44 (37.3%)	0.385	60 (50.8%)	58 (49.2%)	0.050	50 (42.4%)	68 (57.6%)	0.000***
	Above average	101	69 (68.3%)	32 (31.7%)		38 (37.6%)	63 (62.2%)		77 (76.2%)	24 (23.8%)	
Nausea/Vomiting	Below average	172	108 (62.8%)	64 (37.2%)	0.136	76 (44.2%)	96 (55.8%)	0.749	97 (56.4%)	75 (43.6%)	0.360
	Above average	47	35 (74.5%)	12 (25.5%)		22 (46.8%)	25 (53.2%)		30 (63.8%)	17 (36.2%)	
Pain	Below average	137	86 (62.8%)	51 (37.2%)	0.311	66 (48.2%)	71 (51.8%)	0.187	64 (46.7%)	73 (53.3%)	0.000***
	Above average	82	57 (69.5%)	25 (30.5%)		32 (39.0%)	50 (61.0%)		63 (76.8%)	19 (23.2%)	
Dyspnea	Below average	187	124 (66.3%)	63 (33.7%)	0.446	84 (44.9%)	103 (55.1%)	0.902	101 (54.0%)	86 (46.0%)	0.004**
	Above average	32	19 (59.4%)	13 (40.6%)		14 (43.8%)	18 (56.2%)		26 (81.2%)	6 (18.8%)	
Insomnia	Below average	132	82 (62.1%)	50 (37.9%)	0.224	63 (47.7%)	69 (52.3%)	0.275	67 (50.8%)	65 (49.2%)	0.008**
	Above average	87	61 (70.1%)	26 (29.9%)		35 (40.2%)	52 (59.8%)		60 (69.0%)	27 (31.0%)	
Appetite	Below average	160	101 (63.1%)	59(36.9%)	0.266	75 (46.9%)	85 (53.1%)	0.297	78 (48.8%)	82 (51.2%)	0.000***
	Above average	59	42 (71.2%)	17 (28.8%)		23 (39.0%)	36 (61.0%)		49 (83.1%)	10 (16.9%)	
Constipation	Below average	172	112 (65.1%)	60 (34.9%)	0.915	76 (44.2%)	96 (55.8%)	0.749	92 (53.5%)	80 (46.5%)	0.010**
	Above average	47	31 (66.0%)	16 (34.0%)		22 (46.8%)	25 (53.2%)		35 (74.5%)	12 (26.5%)	
Diarrhea	Below average	197	126 (64.0%)	71 (36.0%)	0.213	87 (44.2%)	110 (55.8%)	0.601	108 (54.8%)	89 (45.2%)	0.004**
	Above average	22	17 (77.3%)	5 (22.7%)		11 (50.0%)	11 (50.0%)		19 (86.4%)	3 (13.6%)	
Financial	Below average	107	71 (66.4%)	36 (33.6%)	0.748	43 (40.2%)	64 (59.8%)	0.184	57 (53.3%)	50 (46.7%)	0.167
	Above average	112	72(64.3%)	40 (35.7%)		55 (49.1%)	57 (50.9%)		70 (62.5%)	42 (37.5%)	
Logistical issues											
Transport difficulty	Yes	102	58 (56.9%)	44 (43.1%)	0.014*	37 (36.3%)	65 (63.7%)	0.019*	64 (62.7%)	38 (37.3%)	0.183
	No	107	85 (72.6%)	32 (27.4%)		61 (52.1%)	56 (47.9%)		63 (53.8%)	54 (46.2%)	
Accessibility to medical services	Yes	91	50 (54.9%)	41 (45.1%)	0.007**	34 (37.4%)	57 (62.6%)	0.064	63 (69.2%)	28 (30.8%)	0.000***
	No	128	93 (72.7%)	35 (27.3%)		64 (50.0%)	64 (50.0%)		64 (50.0%)	64 (50.0%)	
Challenges in obtaining E-pass	Yes	42	18 (42.9%)	24 (57.1%)	0.001**	16 (38.1%)	26 (61.9%)	0.335	27 (64.3%)	15 (35.7%)	0.358
	No	177	125 (70.6%)	52 (29.4%)		82 (46.3%)	95 (53.7%)		100 (56.5%)	77 (43.5%)	
Accommodation difficulty	Yes	50	26 (52.0%)	24 (48.0%)	0.025*	16 (32.0%)	34 (68.0%)	0.039*	35 (70.0%)	15 (30.0%)	0.050*
	No	169	117 (69.2%)	52 (30.8%)		82 (48.5%)	87 (51.5%)		92 (54.4%)	77 (45.6%)	
Meeting daily needs	Yes	85	41 (48.2%)	44 (51.8%)	0.000***	23 (27.1%)	62 (72.9%)	0.000***	55 (64.7%)	30 (35.3%)	0.109
	No	134	102 (76.1%)	32 (23.9%)		75 (56.0%)	59 (44.0%)		72 (53.7%)	62 (46.3%)	
Social issues											
Social out-casting	Yes	33	12 (36.4%)	21 (63.6%)	0.000***	16 (48.5%)	17 (51.5%)	0.640	19 (57.6%)	14 (42.4%)	0.958
	No	186	131 (70.4%)	55 (29.6%)		82 (44.1%)	104 (55.9%)		108 (51.1%)	78 (41.9%)	
Isolated from society	Yes	38	16 (42.1%)	22 (57.9%)	0.001**	20 (52.6%)	18 (47.4%)	0.282	22 (57.9%)	16 (41.1%)	0.989
	No	181	127 (70.2%)	54 (29.8%)		78 (43.1%)	103 (56.9%)		105 (58.0%)	76 (42.0%)	
Support from family	Yes	177	120 (67.8%)	57 (32.2%)	0.111	87 (49.2%)	90 (50.8%)	0.007**	102 (57.6%)	75 (42.4%)	0.823
	No	42	23 (54.8%)	19 (45.2%)		11 (26.2%)	31 (73.8%)		25 (59.5%)	17 (40.5%)	
Support from friends	Yes	162	109 (67.3%)	53 (32.7%)	0.298	75 (46.3%)	87 (53.7%)	0.438	92 (56.8%)	70 (43.2%)	0.544
	No	57	34 (59.6%)	23 (40.4%)		23 (40.4%)	34 (59.6%)		35 (61.4%)	22 (38.6%)	
Support from Govt	Yes	125	85 (68.0%)	40 (32.0%)	0.333	62 (49.6%)	63 (50.4%)	0.096	75 (60.0%)	50 (40.0%)	0.487
	No	94	58 (61.7%)	36 (38.3%)		36 (38.3%)	58 (61.7%)		52 (55.3%)	42 (44.7%)	
Support from NGOs	Yes	35	18 (51.4%)	17 (48.6%)	0.060	13 (37.1%)	22 (62.9%)	0.324	25 (71.4%)	10 (28.6%)	0.079
	No	184	125 (67.9%)	59 (32.1%)		85 (46.2%)	99 (53.8%)		102 (54.4%)	82 (44.6%)	
Informational support	Yes	97	56 (57.7%)	41 (42.3%)	0.036*	41 (42.3%)	56 (57.7%)	0.514	58 (59.8%)	39 (40.2%)	0.630
	No	122	87 (71.3%)	35 (28.7%)		57 (46.7%)	65 (53.3%)		69 (56.6%)	53 (43.4%)	
Fear of progression	Below average	143	74 (51.7%)	69 (48.3%)	0.004**	-	-	-	-	-	-
	Above average	76	24 (31.6%)	52 (68.4%)		-	-		-	-	
Global health status	Below average	143	83 (58.0%)	60 (42.0%)	0.983	54 (42.5%)	73 (57.5%)	0.436	-	-	-
	Above average	76	44 (57.9%)	32 (42.1%)		44 (47.8%)	48 (52.2%)		-	-	

*p<0.05,

**p<0.01,

***p<0.001.

Patients with treatment interruption differed significantly in their levels of distress (M(SD) = 3.62(2.5) z = -2.22; p<0.05, r = -0.20) and FOP (M(SD) = 36.10(5.98); z = -2.85; p = <0.01; r = -0.22) from those without treatment interruption. Among the domains of QOL, global health status (M(SD) = 66.56(14.5); z = -2.10; p<0.05; r = -0.14), emotional functioning (M(SD) = 77.71(17.3); z = -4.45; p<0.001; r = -0.30), social functioning (M(SD) = 72.84(24.98); z = -3.07, p<0.001; r = -0.21) and financial concerns (M(SD) = 48.83(35.57); z = -1.99; p<0.05) differed significantly between patients with and without treatment interruption. While neither individual nor coping on the whole differed significantly between the groups, diversion (M(SD) = 14.25(4.79); z = -2.05; p<0.05; r = -0.12), planning (M(SD) = 5.85(1.89); z = -3.04; p<0.01; r = -0.21) and positive focus (M(SD) = 6.35(1.77); z = -3.71; p<0.001; r = -0.25) approaches found to differ significantly between patients with and without treatment interruption. All the independent variables had small effect of the outcome variables (Table 4).

**Table 4:** Mean difference between the treatment status of cancer patients during COVID-19 lockdown and distress, FOP, QOL and cancer coping.

Variables	Treatment status of cancer patients during COIVID 19 Lockdown						
	Interrupted /Delayed (n = 118)		Uninterrupted (n = 101)		*z* value	*Effect Size (r)*	*P*-value
	Mean	SD	Mean	SD			
**Distress**	3.62	2.5	2.96	2.66	-2.22	-0.20	**.026** *
**Fear of Progression**	36.10	5.988	33.87	5.74	-2.85	-0.22	**.004** **
**EORTC QLQ-C30**							
**Functional Scales**							
Global Health Status	66.56	14.5	71.42	16.64	-2.10	-0.14	**.037** *
Physical Functioning	74.44	23.31	77.92	22.79	-1.46	-0.18	.145
Role Functioning	69.71	23.25	68.25	24.27	-0.33	-0.02	.741
Emotional Functioning	77.71	17.3	86.49	18.51	-4.45	-0.30	**.000** **
Cognitive Functioning	82.28	20.85	84	20.55	-0.78	-0.12	.434
Social Functioning	72.84	24.98	81.24	25.35	-3.07	-0.21	**.000** **
**Symptoms Scales**							
Fatigue	18.85	19.83	16.19	19.2	-1.06	-0.12	.289
Nausea/Vomiting	8.18	18.94	12.36	24.65	-0.97	-0.15	.332
Pain	21.87	25.09	18.29	22.89	-0.81	-0.14	.416
Dysponea	6.2	15.66	6.5	17.63	-0.20	-0.01	.842
Insomnia	20.03	28.59	23.08	28.54	-0.98	-0.16	.328
Appetite	16.93	28.46	12.08	28.54	-0.77	-0.12	.439
Constipation	10.16	21.11	12.53	26.58	-0.14	-0.01	.890
Diarrhea	2.53	9.88	7.25	20.32	-1.81	-0.12	.071
Financial	48.83	35.57	39.57	33.3	-1.99	-0.13	**.046** *
**Cancer Coping**							
Individual (total)	32.95	8.73	34.34	8.77	-1.23	-0.13	.218
Coping	11.72	3.05	12.13	3.36	-0.49	-0.03	.621
Positive Focus	6.35	1.77	7.23	1.69	-3.71	-0.25	**.000** **
Diversion	14.25	4.79	15.47	4.53	-2.05	-0.12	**.040** *
Planning	5.85	1.89	6.73	2.03	-3.04	-0.21	**.002** **
Interpersonal	12.63	5.32	13.44	5.62	-1.01	-0.18	.314

Note: SD = Standard Deviation

*p<0.05,

**p<0.01,

***p<0.001.

Age, mode of transport used to commute to the hospital, challenges in meeting daily needs and being out-casted by the society were found to be predictors of distress (ΔR^2^ = 0.02, F = 14.64, *P<0*.*05*). Whereas, education, treatment status, meeting daily needs and support from family were found to be predictors of FOP (ΔR^2^ = 0.02, F = 10.58, *P<0*.*05*). Similarly, social functioning, physical functioning, accessibility to medical services, fatigue, awareness of diagnosis and type of diagnosis were found to predict the global health status of the patients (ΔR^2^ = 0.12, F = 15.19, p*<0*.*05*) (Table 5).

**Table 5:** Multiple regression analyses of distress, fear of progression and global health status.

		B	95% CI for B	SE B	β	
Distress (continuous variable)	Constant	8.97	1.82–4.84	1.26		R^2^ = 0.22 for step4, ΔR^2^ = 0.02 for step 4, F = 14.64 (*P<0*.*05*).
	Age	-0.61	-1.01-(-0.21)	0.20	-.18**	
	Mode of transport	0.66	0.05–1.26	0.31	.13**	
	Social out-casting	-1.70	-2.58-(-0.82)	0.45	-.24***	
	Meeting daily needs	-1.32	-1.94-(-0.63)	0.33	-.24***	
Fear of Progression (continuous variable)	Constant	38.32	33.73–42.92	2.33		R^2^ = 0.29 for step 5, ΔR^2^ = 0.02 for step 5, F = 10.58(*P<0*.*05*).
	Age	1.19	0.16–2.04	0.48	.14*	
	Education	-1.66	-2.45-(-0.87)	0.40	-.26***	
	Treatment Status	-1.82	-3.30-(-0.34)	0.75	-.15*	
	Meeting Daily needs	-2.1	-3.63-(-0.53)	0.79	-.17**	
	Support from family	1.97	0.12–381	0.94	.13*	
Global Health Status (continuous variable)	Constant	24.19	10.67–37.71	6.86		R^2^ = 0.30 for step 6, ΔR^2^ = 0.12 for step 6, F = 15.19 (*P<0*.*05*).
	Type of Diagnosis	1.46	0.20–2.72	0.64	.13*	
	Awareness of diagnosis	6.76	2.00–11.51	2.41	.17**	
	Accessibility to medical services	8.77	5.15–12.54	1.92	.28***	
	Physical Functioning-	0.14	0.06–0.23	0.04	.21**	
	Social Functioning-	0.13	0.1–0.21	0.04	.22**	
	Fatigue	-0.16	-0.26-(-0.01)	0.05	-.19	

Note: CI = Confidence Interval,

* p<0.05,

**P<0.01,

***P<0.001;

B = beta, SE B = unstandardized beta, **β =** Standardized beta.

Demographic and clinical variables which had significant association with distress, FoP and Global health status were included for regression analysis.

## Discussion

More than half of the patients had treatment interruptions and this interruption was in turn associated with distress levels, FOP and global health status among patients. Those patients with treatment interruption were found to have had higher levels of distress owing to their fear that the break in the treatment might lead to worsening of the disease condition, as cancer is an inevitable disease which mandates continued treatment and care. Similarly, treatment interruption had also led to compromised emotional and social functioning among patients.

A major strength of this study is the methodology wherein majority of the data was collected using telephonic survey as the hospital visits were restricted during the lockdown. This eventually resulted in a considerably good sample size for a study carried out during a pandemic lockdown. This is the first study to the best of our knowledge, to attempt studying the psychosocial issues and experiences of cancer patients during a pandemic lockdown. Also, this study compares the psychosocial experiences and issues of patients who have had interruptions in their treatment and those with no interruptions.

Nearly half of the cancer patients in this study were either unable to reach the hospital or were asked to stay at home owing to their compromised immunity and increased risk for COVID-19. Although cancer is an inevitable disease which requires continued care, due to the unprecedented pandemic situation, patients have been disengaged from formal healthcare settings [14].

Patients in this study reported difficulties with accessing transportation as public transportation was curbed and patients had to depend on ambulances/ private vehicles, which was a challenging situation for many patients from rural settings and low socio-economic status. Although travelling for medical reasons were excused in the state, nearly half experiences challenges in finding transportation, and even if found, one-fourth experienced challenges in obtaining e-pass. In view of this, majority of the treatments were postponed or cancelled. In the present study, nearly half of the patients had interruptions in their treatment. In India, around 51,000 cancer surgeries were cancelled between end of March 2020 –May 2020 and the overall cancer care declined by 50% in total [4]. However, recent studies strongly suggest that cancer surgeries cannot be considered elective and should not be postponed and that the guidelines for postponing surgeries are not applicable for cancer surgeries [15].

One area of major concern reported by patients were physical and psychological issues. Pain and fatigue remained as major physical issues reported, irrespective of whether they had treatment interruption or not. Similarly, distress, fear, anxiety, worry and sleep difficulties were found to be common psychological issues prevalent among patients in view of the pandemic situation. This could be attributed to the sudden onset of an uncertain situation and ambiguity in further treatment process, increased risk of acquiring infections and uncertainty regarding prognosis. Surprisingly, beyond physical and functional issues, practical difficulties such as difficulty in accessing medical care, challenges in obtaining e-pass, finding travel and accommodation to proceed with treatment contributed significantly to increased distress among patients. It is clearly evident that such an unprecedented crisis has placed patients and their families in confusion, depriving of necessary support measures. In addition to practical concerns, it is also clearly noted that those patients who were subjected to social out-casting either by their families or by their neighbours experienced high levels of distress and fear of disease progression. This finding emphasises the importance of adequate support from family and society to cancer patients especially at times of such unprecedented health crisis.

Almost half of the patients did not receive adequate informational support on how to manage and deal with the pandemic situation in addition to continuing cancer treatment. This lack of informational support also amplified the levels of distress among patients. Since majority of the healthcare settings were transformed to COVID-19 treatment facilities, cancer patients found it challenging to access medical care pertaining to their needs. Nearly half of the patients in this study reported difficulty in accessing medical facilities at local places. All of these challenges in turn have led to compromised QOL and increased FOP.

Patients with poor familial support, had difficulty in finding transportation and those with treatment interruption during the pandemic were found to have increased FOP. In addition to these, the FOP was highly reported by those with low levels of education. This finding is in line with previous studies stating that highly educated patients may have a greater understanding of the diagnosis however, low level may have clouded information and processing leading to increased unknown fear [16]. All of these clearly indicate that, patients experience excessive fear of uncertainty due to poor informational and communication support. Finally, the logistical issues experienced by patients during pandemic majorly were found to predict distress, fear of progression and the overall health status of the patients.

## Conclusion

More than half of the patients had interruptions in their treatment as a result of COVID-19 lockdown. Overall, cancer patients have had increased physical and psychological concerns as a result of the pandemic situation and its associated changes in the medical care, thereby leading to reduced global health status.

## Implications

Considering the intensity of the issues reported, specific guidelines ought to be framed for providing continued and holistic cancer care for patients during such lockdown. The government need to facilitate guidelines for ensuring continued care for patients with non-communicable diseases, especially cancer. The results suggest a strong felt need for psychosocial care tuned for such pandemic situations especially in oncology setting. Owing to the uncertainty of the situation, appropriate and adequate communication and informational support for patients must be ensured during such times.

## Limitations

The study had one key limitation. The data was collected from only those who reported to the out-patients department during the pandemic lockdown.

## Supporting information

S1 Data Data file of the sample included in the study. (XLSX)
